# A phantom study to determine the optimum size of a single collimator for shortening the treatment time in CyberKnife stereotactic radiosurgery of spherical targets

**DOI:** 10.1120/jacmp.v13i5.3864

**Published:** 2012-09-06

**Authors:** Sudahar Harikrishnaperumal, Gopalakrishna Kurup, Murali Venkatraman, Velmurugan Jagadeesan

**Affiliations:** ^1^ Department of Radiotherapy Apollo Specialty Hospital Chennai 600035 India; ^2^ Department of Medical Physics Anna University Chennai 600025 India

**Keywords:** CyberKnife, treatment time, optimal single collimator, nodes, monitor units, beams

## Abstract

Prolonged treatment execution time is a concern in CyberKnife robotic radiosurgery. Beam reduction and node reduction technique, and monitor unit optimization methods are adopted to reduce the treatment time. Usage of single collimator in the CyberKnife treatment plan can potentially reduce collimator exchange time. An optimal single collimator, which yields an acceptable dose distribution along with minimum number of nodes, beams, and monitor units, can be a versatile alternative for shortening treatment time. The aim of the present study is to find the optimal single collimator in CyberKnife treatment planning to shorten the treatment time with the acceptable dose distribution. A spherical planning target volume PTV1 was drawn in an anthropomorphic head and neck phantom. Plans with same treatment goals were generated for all the 12 collimators independently. $D_{95\%}$ was selected as the prescribing isodose and the prescribed dose was 10 Gy. The plan of the optimal collimator size was evaluated for conformity, homogeneity, and dose spillage outside the target. The optimum collimator size and the target dimensions were correlated. The study was repeated with two other target volumes PTV2 and PTV3 for generalizing the results. Collimator sizes just above the diameter of the spherical PTVs were yielding least number of nodes and beams with acceptable dose distributions. The collimator size of 35 mm is optimum for the PTV1, whose diameter is 31.4 mm. Similarly, 50 mm collimator is optimum for PTV2 (diameter=45.2 mm) and 20 mm collimator is optimum for PTV3 (Diameter=17.3 mm). The total number of monitor units is found to reduce with increasing collimator size. Optimal single collimator is found to be useful for shortening the treatment time in spherical targets. Studies on two clinical targets, (a brain metastasis and a liver metastasis cases) show comparable results with the phantom study.

PACS numbers: 87.55.D, 87.55.de, 87.53.Ly, 87.55.kh, 87.56.nk, 87.55.ne, 87.55.‐x

## I. INTRODUCTION

Stereotactic radiosurgery is a dedicated radiotherapy procedure that is performed with high precision. There are several systems and methods available to perform the radio surgical procedure.^1^, ^2^, ^3^ CyberKnife is an advanced stereotactic radiosurgery treatment unit in which a miniature type linear accelerator is mounted on an industrial robot.^4^, ^5^ The uniqueness of CyberKnife is its multiple noncoplanar beams focusing the target from different points in space called nodes.

Each beam is characterized by any one of the 12 collimator sizes from 5 mm to 60 mm. There are two types of collimator systems available in CyberKnife. One is the variable aperture based IRIS collimator and the other is a set of 12 fixed cone‐type collimators.^(6)^ Treatment execution time of a CyberKnife plan primarily depends upon the number of beams, number of nodes, and the total number of monitor units. Prolonged treatment time is one of the major limitations of CyberKnife treatments and acceptability, as well as clinical utility, of this robotic radiosurgery system will be expanded if we develop measures to reduce the treatment time or ‘patient on couch time’.^7^, ^8^ There are various attempts to reduce treatment time in CyberKnife treatment, including the node reduction technique of Van de Water et al.^(9)^

Apart from the individualized methods, there are few specific tools are provided in the MultiPlan planning system itself to reduce the monitor units and number of beams. Total number of monitor units are optimized using the optimize monitor units (OMU) option, which can be set as a goal during the treatment planning. Similarly for reducing the number of beams, there is an explicit beam reduction option provided in the planning system. This beam reduction can be performed along with an optimization option to preserve the integrity of the treatment plan. Furthermore the selection of a collimator size for an irregular shaped target is a challenging task in CyberKnife as there are 12 choices of collimators. The choice of collimator size can influence the CyberKnife treatment plans.^(10)^ Poll et al.^(8)^ studied the use of optimized multiple collimators for reducing the monitor units in robotic radiosurgery. Usage of single collimator can potentially reduce the collimator exchanging time in fixed cone‐type collimator system. In the present study, an attempt is made to find out the optimal single fixed cone‐type collimator that will yield least number of monitor units, beams, and nodes with acceptable dose distribution. The treatment plans generated for a particular target with 12 single collimators of different size may not have similar dose distributions and treatment parameters. The treatment execution time will be the least for the plan, which has minimum number of nodes, beams, and monitor units. The aim of the present study is to find out the optimum single collimator size that gives shorter treatment time without compromising the treatment goals. Also this study is aimed to correlate the optimal collimator size with the dimension of the target by performing this trial with two other target volumes.

## II. MATERIALS AND METHODS

**Figure 1:**
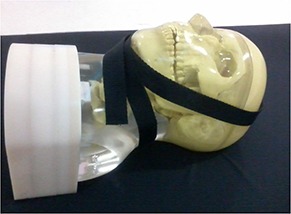
The anthropomorphic head and neck phantom.

An anthropomorphic head neck phantom (Fig. 1) containing a ball cube which is used to perform the end‐to‐end quality assurance for skull tracking and spine tracking in CyberKnife (Accuray Inc., Sunnyvale, CA) is selected for this study. CT scan images of the head and neck phantom are imported into the MultiPlan planning system (Accuray Inc.). The ball inside the phantom is taken as the planning target volume (PTV) and it is named as PTV1. Volume of PTV1 is 16.1 cc.

### A. Treatment planning procedure

The treatment planning started with the alignment. Head and neck phantom is aligned for 6D skull tracking method and number of fractions is taken as one. Sequential optimization is selected for the treatment planning. Initially 5 mm collimator is selected. Then four dose limiting shell volumes are created around the PTV1. First shell covers a radial width of 3 mm around the PTV1 and other shells cover at a radius of 5 mm, 7 mm, and 15 mm radial width around the previous shell. First shell is encompassing the PTV. Limiting dose set for the first shell is 90% of the PTV1 dose. Similarly the limiting dose of 75%, 50%, and 30% of PTV1 dose is set for the second, third, and the fourth shells, respectively. Optimization goal for the PTV1 is set as optimal conformity with 10 Gy dose. Ray tracing algorithm is selected for dose calculations and after optimization was completed, high‐resolution calculations are performed. In the MultiPlan planning system, maximum dose is taken as the default normalization dose. The isodose covering the 95% of the PTV1 is selected for prescription and the prescription dose in this study is 10 Gy.

Planning procedure is repeated for all the 12 collimators as done for the 5 mm collimators. Then volume of the spherical target is enlarged approximately by a factor of 3 and the planning target volume for the second case is named as PTV2. The volume of PTV2 was 48.4 cc. The above described treatment planning procedure was repeated for the PTV2 and dose distribution was calculated. A sphere with approximately one‐sixth (1/6) volume of the PTV1 is taken as the PTV3. The actual volume of PTV3 was 2.7 cc. Twelve plans with 12 different collimator sizes are created for PTV3 also as discussed for the PTV1 and PTV2. The geometrical centre of PTV1, PTV2, and PTV3 are kept the same in this study.

### B. Selection of optimal collimator size for shortened treatment time

The variation of the number of nodes, number of beams, and total monitor units with collimator size is observed for each PTV. From that the collimator sizes, which yield plans with minimum number of nodes, beams, and monitor units are selected. The plans of the selected collimator sizes are analyzed and evaluated for an acceptable dose distribution.

### C. Treatment plan analysis

Treatment plans are analyzed for target coverage and dose spillage outside the target. Factors taken for the analysis of target coverage are $D_{85\%},D_{50\%}$, and $D_{10\%}$ along with the mean, minimum, and maximum target doses. Target dose conformity and homogeneity are also compared using conformity index (CI), new conformity index (nCI) and homogeneity index (HI) parameters. The new conformity index, nCI, is calculated using the formula developed by Paddick^(11)^ and Nakamura et al.,^(12)^ and it is given by the formula:

(1) $$\text{nCI}=\{(\text{TV})\times (V_{\text{RI}})\}/{\left({\text{TV}}_{\text{RI}}\right)}^{2}$$

where *TV* is the volume of the PTV and $V_{\text{RI}}$ is the overall volume covered by the reference prescription isodose. ${\mathrm{TV}}_{\text{RI}}$ is the volume of the PTV covered by the reference prescription isodose. Homogeneity index is the ratio of the maximum dose to the PTV to the reference prescription dose. For analyzing the dose spillage on the normal tissue outside the target, the volume doses of $V_{75\%}$ (cc), $V_{50\%}$ (cc), $V_{30\%}$ (cc), and $V_{20\%}$ (cc) are taken. Here $V_{75\%}$ (cc) indicates the volume in cubic centimeter covered by 75% of the prescribed dose, and it is calculated from the dose volume histogram (DVH). Similarly all the other volumes are also defined in the same way.

## III. RESULTS

Optimal single collimator is selected based on the treatment plans having least number of treatment parameters with reasonable dose distribution. Major treatment parameters, which influence the treatment time, are the number of nodes, beams, and the total number of MUs. The plan analyses based on these parameters are detailed below.

### A. Plan analysis based on number of nodes

**Figure 2:**
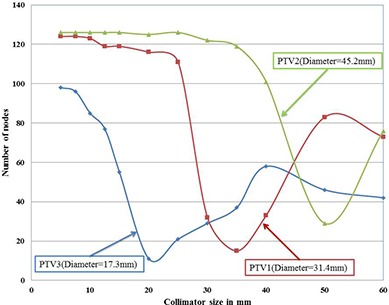
Variation of number of nodes with collimator size for all the three targets.

The number of nodes versus collimator size for all the three targets is shown in Fig. 2. Number of nodes is attaining a low value at a collimator size for each PTV. For PTV1, which is the medium size target with 31.4 mm diameter, the least number of nodes were observed for 35 mm collimator. The least number of nodes were 15. The maximum number of nodes observed for PTV1 was 124 (for both 5 and 7.5 mm collimators). The ratio of maximum and minimum number of nodes was 8.27. Similarly for PTV2, which is the large size target with 45.2 mm diameter, the minimum number of nodes was 29 and it was for 50 mm collimator. For PTV2, maximum number of nodes was obtained for 6 collimators (5 mm, 7.5 mm, 10 mm, 12.5 mm, 15 mm, and 25 mm). The maximum number of nodes was 126. Maximum to minimum ratio of nodes was 4.34. PTV3 is the smallest among all the three spherical targets and has a diameter of 17.3 mm. Only 11 nodes were obtained for 20 mm collimator and this was the least number of nodes for PTV3. Maximum number of nodes for PTV3 was observed for 5 mm collimator and it was 98 nodes. Ratio of maximum to minimum number of nodes was 8.9.

### B. Plan analysis based on number of beams

**Figure 3:**
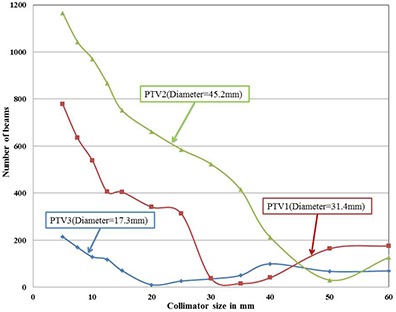
Variation of number of beams with collimator size for all the three targets.

From the observations it is noted that the same collimators, which were resulting least number of nodes, are resulting least number of beams, too. The graph between the number of beams and collimator size for all the three targets is shown in Fig. 3. For PTV1, PTV2, and PTV3, the collimators 35 mm, 50 mm and 20 mm were showing minimum number of beams, respectively. The least numbers of beams were 16, 31, and 11 for PTV1, PTV2, and PTV3, respectively. Maximum numbers of beams for all the three targets were from the smallest 5 mm collimator only. The maximum number of beams were 778, 1165, and 215 for PTV1, PTV2, and PTV3, respectively.

### C. Plan analysis based on number of monitor units

**Figure 4:**
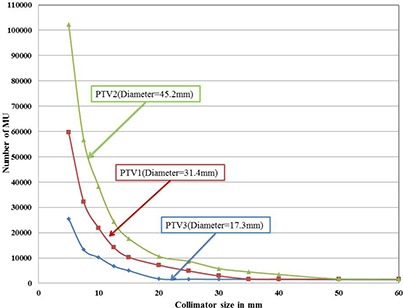
Variation of number of monitor units with collimator size for all the three targets.

Total numbers of MUs of the treatment plans for different collimators are not showing any optimal minimum. However the number of MUs decreases with increase in the collimator size. Variation of number of MU with collimator size is shown in Fig. 4. For all the three targets 5 mm collimator was resulting in maximum number of MUs, while 60 mm was resulting minimum number of MUs. Minimum and maximum MUs for PTV1, PTV2, and PTV3 was 1557, 1679, 1426, and 59663, 102297, 25524, respectively. Based on the above analyses, 35 mm, 50 mm, and 20 mm collimators are showing least number of nodes and beams for PTV1, PTV2, and PTV3, respectively. For declaring these collimators as the optimal single collimators, the treatment plans of these collimators must be evaluated for reasonable dose distribution. The plan evaluation is described below.

### D. Plan evaluation for the optimal collimators

Analysis of the target dose distributions of PTV1, PTV2, and PTV3 is given in Tables 1, 2, and 3, respectively. Dose coverage is similar in all the treatment plans, but they are differing in the conformity index. However, the plans of the above selected collimators of the respective targets are showing better dose coverage and acceptable conformity and homogeneity indices. In the treatment plan of PTV1 with 35 mm collimator, the conformity index (CI) and homogeneity index (HI) was 1.08 and 1.11, respectively. Similarly for PTV2 with 50 mm collimator, CI and HI was 1.16 and 1.18. Treatment plan with 20 mm collimator for PTV3 showed CI and HI of 1.20 and 1.12. Dose was prescribed to the isodose covering 95% volume of the target. $D_{85\%}$, $D_{50\%}$, and $D_{10\%}$ are shown along with the target minimum, maximum, and mean doses in Tables 1–3.

**Table 1:** PTV1 target dose distribution for all the 12 collimators.

		*Target Coverage ‐ PTV1* (Diameter=31.4 mm)								
*Collimator Size (mm)*	*Isodose Covering the 95% Volume (%)*	*CI*	*nCI*	*HI*	*Minimum Dose (cGy)*	*Maximum Dose (cGy)*	*Mean Dose (cGy)*	$D_{85\%}$ *(cGy)*	$D_{50\%}$ *(cGy)*	$D_{10\%}$ *(cGy)*
5	91	1.06	1.11	1.10	941.5	1098.9	1030.5	1011.0	1033.0	1055.0
7.5	92	1.04	1.09	1.09	930.4	1087.0	1032.9	1021.7	1032.6	1054.4
10	92	1.06	1.10	1.09	872.7	1087.0	1037.5	1021.7	1043.5	1054.4
12.5	90	1.09	1.15	1.11	906.1	1111.1	1051.7	1022.2	1055.6	1077.8
15	93	1.04	1.09	1.08	955.8	1075.3	1042.1	1021.5	1043.0	1064.5
20	83	1.68	1.78	1.20	775.1	1204.8	1141.3	1108.4	1156.6	1192.8
25	91	1.07	1.13	1.10	649.8	1098.9	1048.9	1022.0	1055.0	1087.9
30	75	1.27	1.33	1.33	822.7	1333.3	1197.8	1106.7	1213.3	1293.3
35	90	1.04	1.08	1.11	960.0	1111.1	1057.2	1022.2	1055.6	1100.0
40	91	1.06	1.09	1.10	984.6	1098.9	1080.3	1022.0	1055.0	1087.9
50	88	1.12	1.18	1.14	947.3	1136.4	1069.9	1022.7	1068.2	1125.0
60	92	1.32	1.38	1.09	983.0	1087.0	1044.8	1021.7	1043.5	1076.1

**Table 2:** PTV2 target dose distribution for all the 12 collimators.

		*Target Coverage ‐ PTV2* (Diameter=45.2 mm)								
*Collimator Size (mm)*	*Isodose Covering the 95% Volume (%)*	*CI*	*nCI*	*HI*	*Minimum Dose (cGy)*	*Maximum Dose (cGy)*	*Mean Dose (cGy)*	$D_{85\%}$ *(cGy)*	$D_{50\%}$ *(cGy)*	$D_{10\%}$ *(cGy)*
5	86	1.09	1.14	1.16	809.03	1066.20	1162.79	1034.88	1069.77	1104.65
7.5	90	1.04	1.08	1.11	936.82	1041.45	1111.11	1022.22	1044.44	1066.67
10	91	1.03	1.06	1.10	937.37	1046.50	1098.90	1021.98	1043.96	1065.93
12.5	91	1.04	1.07	1.10	802.50	1052.07	1098.90	1032.97	1054.95	1076.92
15	92	1.01	1.07	1.09	942.56	1042.00	1086.96	1021.74	1043.48	1065.22
20	92	1.03	1.06	1.09	949.98	1050.66	1086.96	1021.74	1054.95	1076.09
25	78	1.76	1.85	1.28	787.22	1203.59	1282.05	1166.67	1230.77	1269.23
30	88	1.16	1.22	1.14	822.75	1078.28	1136.36	1034.09	1090.91	1125.60
35	83	1.09	1.15	1.20	911.94	1111.89	1204.84	1036.14	1120.48	1192.77
40	80	1.03	1.10	1.25	865.67	1145.27	1250.00	1050.00	1162.50	1237.50
50	85	1.10	1.16	1.18	933.43	1086.65	1176.47	1023.53	1094.12	1152.94
60	85	1.05	1.11	1.18	964.38	1076.96	1176.47	1023.53	1070.59	1141.18

**Table 3:** PTV3 target dose distribution for all the 12 collimators.

		*Target Coverage ‐ PTV3* (Diameter=17.3 mm)								
*Collimator Size (mm)*	*Isodose Covering the 95% Volume (%)*	*CI*	*nCI*	*HI*	*Minimum Dose (cGy)*	*Maximum Dose (cGy)*	*Mean Dose (cGy)*	$D_{85\%}$ *(cGy)*	$D_{50\%}$ *(cGy)*	$D_{10\%}$ *(cGy)*
5	84	1.34	1.41	1.19	894.04	1069.97	1190.48	1023.81	1071.43	1130.95
7.5	90	1.18	1.23	1.11	960.36	1041.58	1111.11	1022.22	1044.44	1077.78
10	79	1.88	1.98	1.27	821.73	1151.95	1265.82	1101.27	1164.56	1262.53
12.5	85	1.75	1.85	1.18	728.70	1105.58	1176.47	1082.35	1117.65	1141.18
15	88	1.26	1.32	1.14	934.03	1067.75	1136.36	1034.09	1068.18	1102.27
20	89	1.13	1.20	1.12	915.79	1067.61	1123.60	1033.71	1078.65	1112.36
25	93	1.07	1.11	1.08	978.13	1040.10	1075.27	1021.51	1043.01	1064.52
30	93	1.09	1.13	1.08	978.85	1041.67	1075.27	1010.75	1043.01	1054.52
35	95	1.12	1.15	1.05	990.44	1026.96	1052.63	1021.51	1043.01	1034.52
40	95	1.97	2.11	1.05	986.26	1026.46	1052.63	1010.53	1031.58	1042.11
50	95	4.00	4.18	1.05	990.44	1026.47	1052.57	1010.52	1021.05	1042.11
60	97	3.43	3.49	1.03	995.56	1018.22	1030.93	1010.31	1020.62	1030.93

**Figure 5:**
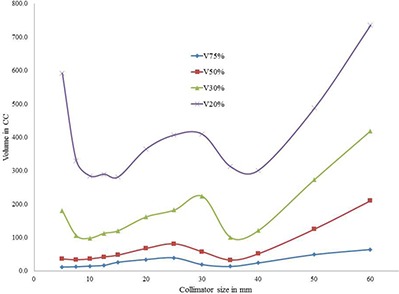
Analysis of dose spillage on normal tissue surrounding the PTV1.

**Figure 6:**
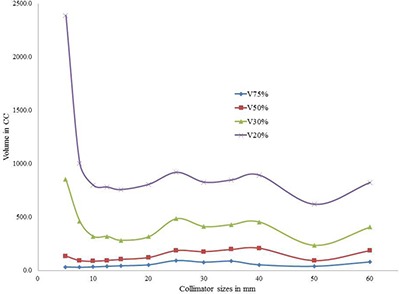
Analysis of dose spillage on normal tissue surrounding the PTV2.

**Figure 7:**
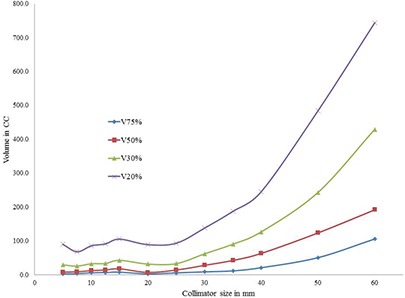
Analysis of dose spillage on normal tissue surrounding the PTV3.

**Figure 8:**
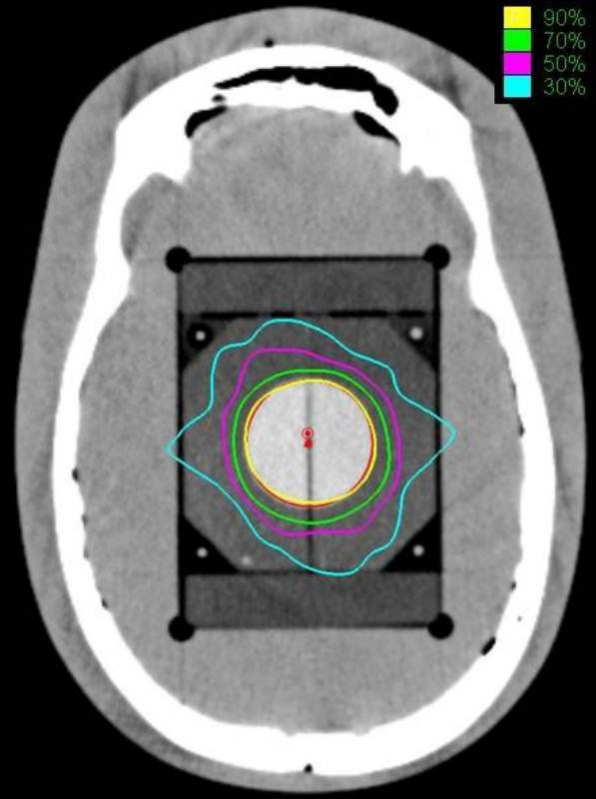
An example dose distribution by the optimal collimator (35 mm collimator) in the axial CT image for PTV1.

For an acceptable plan, dose spillage outside the target must be within the tolerance of the surrounding organs. In the present study, the dose spillage outside the target due to the selection of single collimators was compared. For the medium type target PTV1, dose spillage on normal tissue outside the target was higher for both smaller and larger collimators (Fig. 5). However, for the larger size target PTV2, dose spillage is larger for smaller collimators than the larger collimators (Fig. 6). Similarly, for the smaller size target PTV3, dose spillage is larger for larger collimators than the smaller collimators (Fig. 7). It is interesting to note that for all the three targets, the $V_{75\%}$, $V_{50\%}$, $V_{30\%}$, and $V_{20\%}$ (cc) values were attaining a local minimum for the selected collimators which were yielding the least number of treatment parameters. The axial dose distribution for 35 mm collimator corresponding to the PTV1 is shown in Fig. 8.

## IV. DISCUSSION

Robotic radiosurgery (CyberKnife) is the recent radiation therapy delivery system which can deliver precise high‐radiation dose to the target. Hence, CyberKnife can deliver patient‐friendly short course hypofractionated radiation therapy or ‘fractionated radiosurgery’ with high dose to the target and minimal dose to the adjacent critical structures. Major advantage of this ‘frameless’ radiosurgery system is that it can deliver high‐dose radiation even in moving structures such as in lung and liver. However, the main criticism of this multiple pencil beam radiation therapy delivery system is its long treatment time. Prolonged treatment time has an impact on moving structures, especially lung and liver as these structures move with respiration and can move up to 3 to 4 cm with respiration. Prolonged treatment time in such situation is challenging task to track the ‘fiducials’ and most of the time uncomfortable for the patient to cooperate for long time. Methods to shorten the treatment time without compromising on the dose distribution parameters are the way forward to enhance the utility of CyberKnife. As discussed earlier, there are several methods to reduce the number of beams, nodes, and monitor unit to reduce the treatment time. Poll et al.^(8)^ tried to reduce the treatment time by using combinations of multiple collimators. In that study, the two cone (collimator) system scores over single cone in MU reduction. However in that study, all the 12 single collimators were not taken for one cone plan to compare with the two cone plans. CyberKnife planning system enables to minimize beams by eliminating the beams with minimal monitor unit contribution.

In the present study, our major focus is to establish the appropriate single collimator which can deliver approvable dose distribution (target coverage and organ sparing) with minimal treatment time. Our present study showed that collimator sizes, which are just above the diameter of the spherical target, are producing quotable lower number of nodes and beams. However, the number of monitor units is decreasing with increasing collimator size but increasing with target size. Inappropriate choice of single collimator for PTV1 can yield 126 nodes and 778 beams, while the optimal 35 mm collimator yields only 15 nodes and 16 beams with acceptable conformity index and dose distribution. Similarly, for PTV2 and PTV3, an appreciable node and beam reduction was observed with acceptable dose distributions. Maximum MU for PTV1 was obtained from 5 mm collimator and it was 59663, whereas with optimal 35 mm collimator it was 1706. This suggests that inappropriate choice of single collimator for PTV1 can increase total MU by a factor of 34.9. This factor for PTV2 is 58.8 (MU max=102297 (5 mm collimator), MU optimum =1739 (50 mm collimator)). Similarly for PTV3, MU reduction factor by the optimum collimator of 20 mm for PTV3 is 14.2 (maximum MU was 25525 and with optimum collimator 1798). Reduction factor is higher with increase in the target size. Studies by Poll et al.^(8)^ show a maximum of 56% MU reduction for two cone plans over one cone plans. Novel node reduction technique used by van de Water et al.^(9)^ reduces the number of nodes but not the number of beams and the number of MU. However, the overall treatment time is reduced appreciably by the node reduction. The optimal collimators selected in the present study for the three different target volumes are yielding minimum number of nodes and beams, and reasonably lower MUs with acceptable dose distributions. Based on these evidences, it can be stated that the shortening of treatment time is very much possible with the optimal single collimators.

We have tried to have a clinical correlation and hence taken two clinical cases to evaluate the present hypothesis in clinical situation. First case (Case 1, intracranial) a brain metastasis and another case (Case 2, extracranial) a liver metastasis were contoured and planned for all the 12 single collimators. The target coverage analysis along with the number of nodes, beams, and monitor units for all the 12 collimator plans of Case 1 and Case 2 are shown in Tables 4 and 5, respectively. Diameter of the cranial target (Case 1) is about 23.5 mm. From the table it can be noted that the least treatment parameters are obtained for 25 mm collimator and hence 25 mm is the optimum collimator. This result favors the proposed hypothesis of existence of optimal collimator for spherical targets.

**Table 4:** Treatment parameters and target dose coverage analysis of Case 1 (cranial target) for all the 12 collimator plans.

	*Total Number of*				*Target Coverage Analysis for Clinical Case 1 (brain metastasis target of* diameter=23.5 mm)								
*Collimator Size (mm)*	*Nodes*	*Beams*	*Monitor Units*	*Isodose Covering 95% Volume (%)*	*CI*	*nCI*	*HI*	*Minimum Dose (cGy)*	*Maximum Dose (cGy)*	*Mean Dose (cGy)*	$D_{85\%}$ *(cGy)*	$D_{50\%}$ *(cGy)*	$D_{10\%}$ *(cGy)*
5	96	321	95747	67	1.45	1.51	1.49	1661.9	3358.2	2559.6	2384.3	2552.2	2787.3
7.5	107	234	57147	81	1.42	1.48	1.23	2061.1	2777.8	2439.6	2333.3	244.4	2583.3
10	94	185	38867	83	1.43	1.48	1.20	2132.9	2710.8	2439.3	2331.3	2439.8	2575.3
12.5	84	143	25972	84	1.38	1.44	1.19	2124.2	2678.6	2420.8	2303.6	2410.7	2544.6
15	74	127	17896	83	1.42	1.48	1.20	2059.2	2710.8	2460.5	2358.4	2466.9	2575.3
20	67	94	11606	83	1.55	1.60	1.20	1841.5	2710.8	2471.8	2358.3	2466.9	2602.4
25	20	21	6708	81	1.30	1.35	1.23	1986.9	2777.8	2533.3	2388.9	2555.6	2694.4
30	23	27	4448	87	1.23	1.27	1.15	2109.0	2586.2	2392.9	2301.7	2379.3	2508.6
35	31	36	3930	91	1.27	1.32	1.10	2156.0	2472.5	2352.4	2299.5	2348.9	2423.1
40	31	35	3857	93	1.26	1.30	1.08	2179.1	2419.4	2335.9	2274.2	2322.6	2395.2
50	35	45	3749	93	2.24	2.32	1.08	2042.2	2419.4	2349.3	2298.4	2346.7	2395.1
60	31	37	3579	91	5.39	5.70	1.09	2212.2	2471.4	2378.1	2299.5	2373.6	2447.8

**Table 5:** Treatment parameters and target dose coverage analysis of Case 2 (liver target) for all the 12 collimator plans.

	*Total Number of*				*Target Coverage Analysis for Clinical Case 2 (brain metastasis target of* diameter=42.5 mm)								
*Collimator Size (mm)*	*Nodes*	*Beams*	*Monitor Units*	*Isodose Covering 95% Volume (%)*	*CI*	*nCI*	*HI*	*Minimum Dose (cGy)*	*Maximum Dose (cGy)*	*Mean Dose (cGy)*	$D_{85\%}$ *(cGy)*	$D_{50\%}$ *(cGy)*	$D_{10\%}$ *(cGy)*
5	59	174	‐	3	‐	‐	‐	‐	‐	‐	‐	‐	‐
7.5	59	227	‐	24	‐	‐	‐	‐	‐	‐	‐	‐	‐
10	60	328	‐	63	‐	‐	‐	‐	‐	‐	‐	‐	‐
12.5	77	469	105816	86	1.16	1.21	1.16	3278	4535	4103	4036	4127	4172
15	75	353	73887	88	1.15	1.21	1.14	3073	4432	4086	3989	4122	4166
20	73	281	50470	86	1.39	1.46	1.16	3342	4535	4219	4081	4263	4353
25	72	242	34897	84	1.76	1.80	1.19	3008	4643	4360	4225	4411	4504
30	67	201	27484	86	1.66	1.73	1.16	2874	4535	4283	4127	4308	4444
35	49	92	22426	83	1.57	1.63	1.20	3359	4699	4367	4088	4370	4652
40	29	33	11131	84	1.18	1.25	1.19	3204	4643	4333	4086	4364	4596
50	23	28	10008	88	1.40	1.47	1.14	3569	4432	4192	4033	4210	4388
60	25	44	9944	89	1.51	1.57	1.12	3507	4382	4174	4031	4207	4338

**Figure 9:**
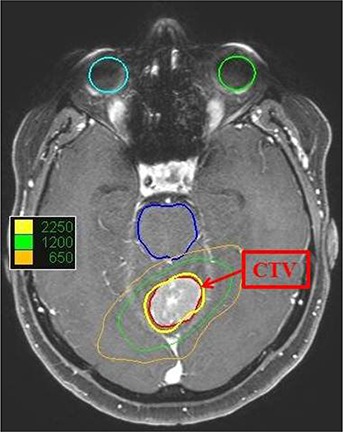
An axial dose distribution in optimal single collimator plan of clinical Case 1 (brain).

**Figure 10:**
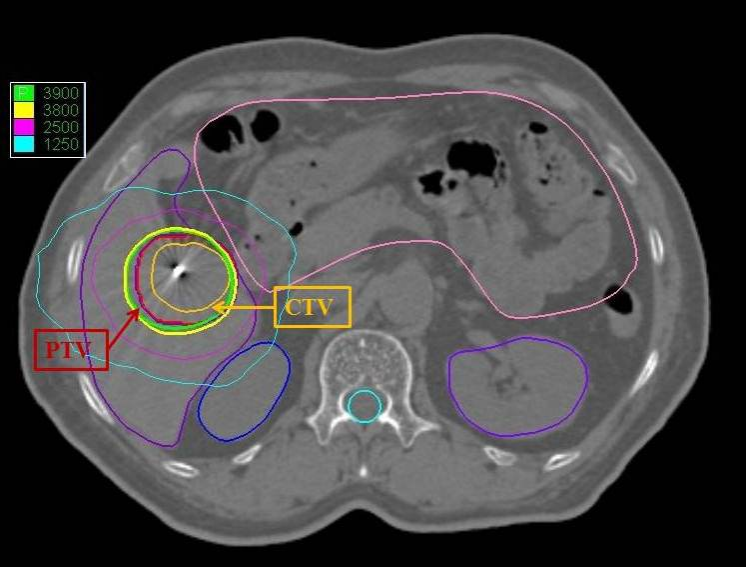
An axial dose distribution in optimal single collimator plan of clinical Case 2 (liver).

Similarly for Case 2 (liver target), the diameter is about 42.5 mm and the optimal collimator is found out as 50 mm. These results are also supporting the findings of the present study. However, while performing the optimization with the set goals, the smaller collimators namely 5 mm, 7.5 mm, and 10 mm, were not yielding a complete solution. The 95% target volume was covered by 3%, 24%, and 63% isodose lines for 5 mm, 7.5 mm, and 10 mm collimators, respectively. The target size could be a possible reason for this. However, in the skull phantom study, the diameter of PTV2 was 45.2 mm; still it was possible to get a reasonable dose distribution. The reason for this could be the “head path” which has more degrees of freedom than the “body path” used for extracranial sites like the liver target. Here “path” is the set of predefined beams which are site‐specific. The number of nodes and beams of the plans of these three smaller collimators (5 mm, 7.5 mm, and 10 mm) are very much higher than the optimal collimator (50 mm). Though this problem exists for smaller collimators, it was possible to get reasonably acceptable dose distributions with other collimators. An axial dose distribution in optimum collimator plans of the Case 1 and Case 2 are shown in Figs. 9 and 10, respectively. Evaluation of the present hypothesis with systematic extension in targets of different shapes and sizes may extend the present findings.

## V. CONCLUSIONS

In summary, it may be stated that shortening of treatment time is very much possible with the optimal single collimators in spherical targets. Single collimator just above the diameter of the spherical target may be the optimal collimator which will deliver acceptable dose distribution with minimal nodes, beams, and MUs which, in turn, will reduce the treatment time. However, there is need for a systematic extension and validation of the present hypothesis for possible CyberKnife clinical planning practice guideline applicable in different tumor shapes and sizes.
